# Icariin-conditioned serum combined with chitosan attenuates cartilage injury in rabbit knees with osteochondral defect

**DOI:** 10.1186/s13018-023-03607-w

**Published:** 2023-02-21

**Authors:** Juntao Zhang, Fangyang Fan, Chao Zhang, Aifeng Liu, Man Shang, Lin Meng

**Affiliations:** 1 Academy of Medical Engineering and Traditional Medicine, Tianjin University, Weijin Road, Nankai District, Tianjin, China; 2 Orthopedics Department, National Clinical Research Center for Chinese Medicine Acupuncture and Moxibustion, The First Teaching Hospital of Tianjin University of Traditional Chinese Medicine, Tianjin, China; 3 School of Basic Medical Sciences, Tianjin Medical University, Qixiangtai Road, Heping District, Tianjin, China

**Keywords:** Osteoarthritis, Icariin, Chitosan, Cartilage repair, Subchondral bone

## Abstract

**Background:**

Knee osteoarthritis (KOA) is one of the most common degenerative diseases. Its development is closely related to cartilage injury and subchondral bone remodeling homeostasis. In the present study, we combined icariin-conditioned serum (ICS) with thiolated chitosan (CSSH), a material widely used in tissue engineering for cartilage repair, to demonstrate its effect on the repair of cartilage damage and abnormal subchondral remodeling.

**Methods:**

New Zealand rabbits were undergoing surgery for cartilage defect, and joint cavity injection was performed in each group with 0.5 mL normal saline (NS), ICS, CSSH and ICS-CSSH in the right joint every week for five times. Positioning performance was observed using VICON motion capture system. Glycosaminoglycans (GAG) secretion of articular fluid was assessed. Osteoarthritis Research Society International (OARSI) score and immunohistochemical (IHC) analysis including H&E, Safranin O and collagen II staining were employed to evaluate the morphologic repair of cartilage and subchondral bone. mRNA expression of COL2A1, MMP13 and ADAMTS5 was detected in chondrocytes from injury area.

**Results:**

ICS combined with CSSH attenuated cartilage injury and abnormal subchondral remodeling in rabbits with KOA. ICS and CSSH groups showed slight improvement in positioning performance, while ICS-CSSH group exhibited better positioning performance. ICS-CSSH group showed increased GAG secretion of articular fluid and expression of COL2A1 in articular chondrocytes. Furthermore, both macroscopic observation and IHC analysis showed femoral condyle in ICS-CSSH rabbits were repaired with more native cartilage and subchondral bone regeneration.

**Conclusions:**

ICS combined with CSSH could promote the repair of osteochondral defect and stabilize subchondral bone remodeling in rabbit knees.

**Supplementary Information:**

The online version contains supplementary material available at 10.1186/s13018-023-03607-w.

## Background

As one of the most common degenerative diseases, knee osteoarthritis (KOA) is closely related to the degeneration of cartilage [1, 2]. Although its pathogenesis is unclear, it is noteworthy that cartilage and subchondral bone homeostasis plays an important role in the progression of KOA. Cartilage is an avascular tissue, and the absence of blood vessels is considered a key feature in the homeostasis of permanent cartilage. Intact cartilage and its microenvironment provide balanced support for the stabilization of joints. Subchondral bone consists of subchondral bone plate, cancellous bone and other structures, and contains blood vessels, nerves and bone marrow [3, 4]. Therefore, it is very essential for the metabolic processes and nutritional support of articular cartilage. It plays an important role in the protection of articular cartilage by providing support and shock absorption for normal joints, protecting the articular cartilage from excessive impact forces and maintaining the shape of the joint [5, 6]. Under normal conditions, subchondral bone maintains a dynamic balance of osteogenesis and osteoclastic resorption. Normal subchondral bone remodeling is an important part of maintaining stability and renewing bone mass [7]. Therefore, maintaining cartilage intact, subchondral bone remodeling homeostasis may serve as a new idea to slow down the progression of KOA.

Icariin is a prenylated flavonol glycoside extracted from a traditional Chinese medicine Epimedium herb. It is the main bioactive component of Epimedium herb and has been shown more effective than other flavonoid compounds in promoting chondrocyte vitality and articular cartilage repair [8, 9]. In addition, it has been found that icariin not only promotes chondrocyte proliferation and differentiation, but also integrates with subchondral bone [8]. Because of its osteogenic, chondrogenic, and anti-inflammatory effects, icariin may be a natural drug for maintaining subchondral bone remodeling homeostasis and slowing the progression of KOA in early stage.

Autologous conditioned serum (ACS) is an innovative therapy that has been assayed in experiments [10, 11]. ACS is considered to be used in the improvement of tissue regeneration and reduction in degenerative mechanisms. Some clinical trials have demonstrated its beneficial effect in KOA [12, 13]. It has been applied in intra-articular injections for patients with a risk of osteoarthritis development in Frizziero' research [14]. Conditioned serum has been used intra-articularly for osteoarthritic patients possibly attributing to its enrichment in anti-inflammatory proteins and growth factors in regenerative medicine applications [15]. Based on this rationale, rabbit conditioned serum with icariin was prepared in our previous research and has showed significant proliferation effects on primary rabbit chondrocytes in vitro and osteochondral defect models in vivo [16].

Combination treatment with bioactive materials is a burgeoning area in tissue engineering and regenerative medicine. Chitosan is a deacetylated derivative from chitin with good biocompatibility and is widely used in therapies including wound healing, vascular tissue engineering and dental implants [17–19]. In addition, one study reported that hydroxyapatite-grafted-chitosan promoted subchondral bone remodeling, osteogenesis and chondrogenesis [20]. It is promising in the treatment of osteoarthritis repair as a base for osteoplastic materials with its good biocompatibility [13]. Thus, it is proposed that combination of ICS with chitosan will exhibit facilitating the repair of osteochondral defect and reduction in subchondral bone osteoporosis.

In the present research, combination of ICS with chitosan is applied in the treatment of osteoarthritis model in rabbits to elucidate its role in cartilage regeneration, osteochondral defect repair and subchondral bone remodeling homeostasis.

## Materials and methods

### Animals and osteoarthritis model

New Zealand White Rabbits with age of 12 weeks were obtained from Charles River Laboratories. Rabbits were maintained under specific pathogen-free (SPF) and temperature-controlled environment with free access to food and water. Rabbits with the weight about 3 kg were used for the establishment of osteoarthritis model. The surgery was conducted in rabbit knees as previously reported. The cartilage defect was made on the femoral condyle with a diameter of 4 mm and a depth of 3 mm [21]. Rabbits were allowed to move freely after the operation and enrolled in further experiments with no infective complications. All animal experiments were performed in accordance with the Declaration of Helsinki of the World Medical Association and approved by Ethics Committee of Tianjin University of Traditional Chinese Medicine (TCM-LAEC20170026).

### ICS preparation

New Zealand white rabbits were gavaged with icariin (Shanghai Ronghe, Lot: 160,602, purifity ≥ 98%) for 1 week at the dosage of 0.94 g/kg every day [16]. The gavage dose of icariin was calculated according to the equivalent dose of icariin in human. In detail, human dose of Icariin 10 g was converted to rabbit dose according to body surface area. Proportion of dose conversion between human (70 kg) and rabbit (1.5 kg) is 14.2 [21]. The rabbit gavage dose of icariin was calculated according to the proportional conversion. Two hours after the last administration, rabbits were anesthetized using urethane and circulating blood were drawn from aorta abdominalis to prepare ICS. Serum was collected by centrifugation of 2000 g for 10 min. ICS was prepared after freezing by FD5-2.5 Freeze Dryer (SIM, Newark, USA). The ICS powder is easily dissolved in normal saline and chitosan solution.

### Synthesis of thiolated chitosan (CSSH)

Chitosan (Sangon, Shanghai, China) with the weight of 500 mg was dispersed in 46 ml distilled water and stirred for 5 min, followed by further stirring for 30 min with addition of 348.6 mg HOBT (Sangon, Shanghai, China). NAC with the weight of 842 mg was added and stirred for 5 min after the previous solution become limpidly. EDC-HCl solution (1978.5 mg of EDCI-HCl dissolved in 4 mL of distilled water) was added, and then, the pH was adjusted at about 5.

The reaction product was dialyzed with molecular weight cut off at 7000 in the sequential solution of 5 mM hydrochloric acid and 2 μM EDTA for 3 days, the solution of 5 mM hydrochloric acid, 2 μM EDTA and 0.1% NaCl for 2 days, the solution of 5 mM hydrochloric acid for 1 day, and finally, the distilled water for 1 day. The entire dialysis process was conducted at 4℃ in dark.

### Animal treatment

All rabbits with osteoarthritis were randomly divided into four groups including NS (normal saline), ICS, CSSH and ICS-CSSH. Six rabbits in each group were injected intra-articularly with 0.5 ml NS, ICS, CSSH and ICS combined with CSSH in the right knee joint every week, respectively. The administration was started at the beginning of third week after surgery and lasted for five weeks. All animals were executed with overdose sodium pentobarbital (MTC Pharmaceuticals, Ontario, Canada) 12 weeks after surgery. Joint fluid was extracted from the knee cavity and collected by centrifugation at 5000 g for 10 min. The content of glycosaminoglycans (GAG) in the articular fluid was detected according to the manufacturer’s instructions (MyBioSource, Inc., CA, USA). The optical density (OD value) was measured using a microplate reader set to 450 nm. Knee joints were collected for macroscopic evaluation and histological analysis.

### Positioning performance measurement

The motion data were collected using Vicon Motion System (Vicon Motion Systems Ltd, Oxford, UK) on the rabbits before and 12 weeks after surgery. The motion data were quantified at 200 Hz with a 15-camera motion capture system through reflective makers stuck on the rabbit. In detail, the reflective markers were placed at 9 points including rabbit phalanges, joint of tarsal bone and the tibia, joint of the tibia and femur and termination of femur, and the middle of spine. The joint angles of ankles when rabbit jumping from slope top to the bottom were analyzed. Angle change of motion was calculated according to the angle data collected before and 12 weeks after surgery.

### Observation of osteoarthritic joint and pathologic evaluation of OA in rabbit knees

Knee joints were collected from rabbits 12 weeks after the surgery and photoed to exhibit the repair of cartilage defect. Osteoarthritis Research Society International (OARSI) pathology assessment system was applied for the evaluation of animal models of OA [22]. The score evaluated by OARSI system represented a combined assessment which is based on both the severity (grade) and extent (stage) of OA in the articular cartilage. The recommended score was an index of combined grade and stage, calculated by the simple formula: score = grade * stage.

### Histopathologic analysis of femoral condyle of rabbits with osteoarthritis

The joint tissues were fixed in 4% paraformaldehyde for 48 h and decalcified in 10% EDTA-Na_2_ for 3 weeks, followed by dehydration in sequential ethanol. Samples were embedded in paraffin and sliced into 5-μm sections. Sample slides were then stained by Hematoxylin and eosin (H&E), safranin O and anti-collagen II antibody, respectively. Mankin’s histopathology grading system based on microscopic evaluation of H&E histology was used for grading OA progression in samples from each group [23].

### RNA extraction and quantitative real-time PCR (qPCR)

Tissue fractions from injured articular surface were collected, minced in liquid nitrogen. Total RNA was extracted using TriZOL. cDNA was reversely transcribed using One-Step cDNA Synthesis SuperMix (Transgen, Beijing, China). Expression of COL2A1, MMP13, ADAMTS5 was evaluated from quantitative real-time PCR and calculated using 2^−△△t^ methods. Primers for qPCR were listed. β-actin (forward):cgcatgcagaaggagatcac, β-actin (reverse): cgactcgtcatactcctgct, COL2A1 (forward):ccaagggagagcaaggagaa, COL2A1 (reverse): cctttggggccttcttttcc, MMP13 (forward):ctgcccctcctcaacagtaa, MMP13 (reverse): cctgtcacctctaagccgaa, ADAMTS5 (forward): attcttgcaacggacccaac, ADAMTS5 (reverse):tccctttgctaacttccggt.

### Statistics

Results were calculated as mean ± s.e.m. using Prism 7 software. Significance between two groups was determined using the Student’s *t* test (unpaired two-tailed, unequal variance). It was considered statistically significant when *P* < 0.05. *N* numbers are indicated in the figure legends.

## Results

### Exhibition of osteoarthritis model procedure and treatment solutions

**Fig. 1:**
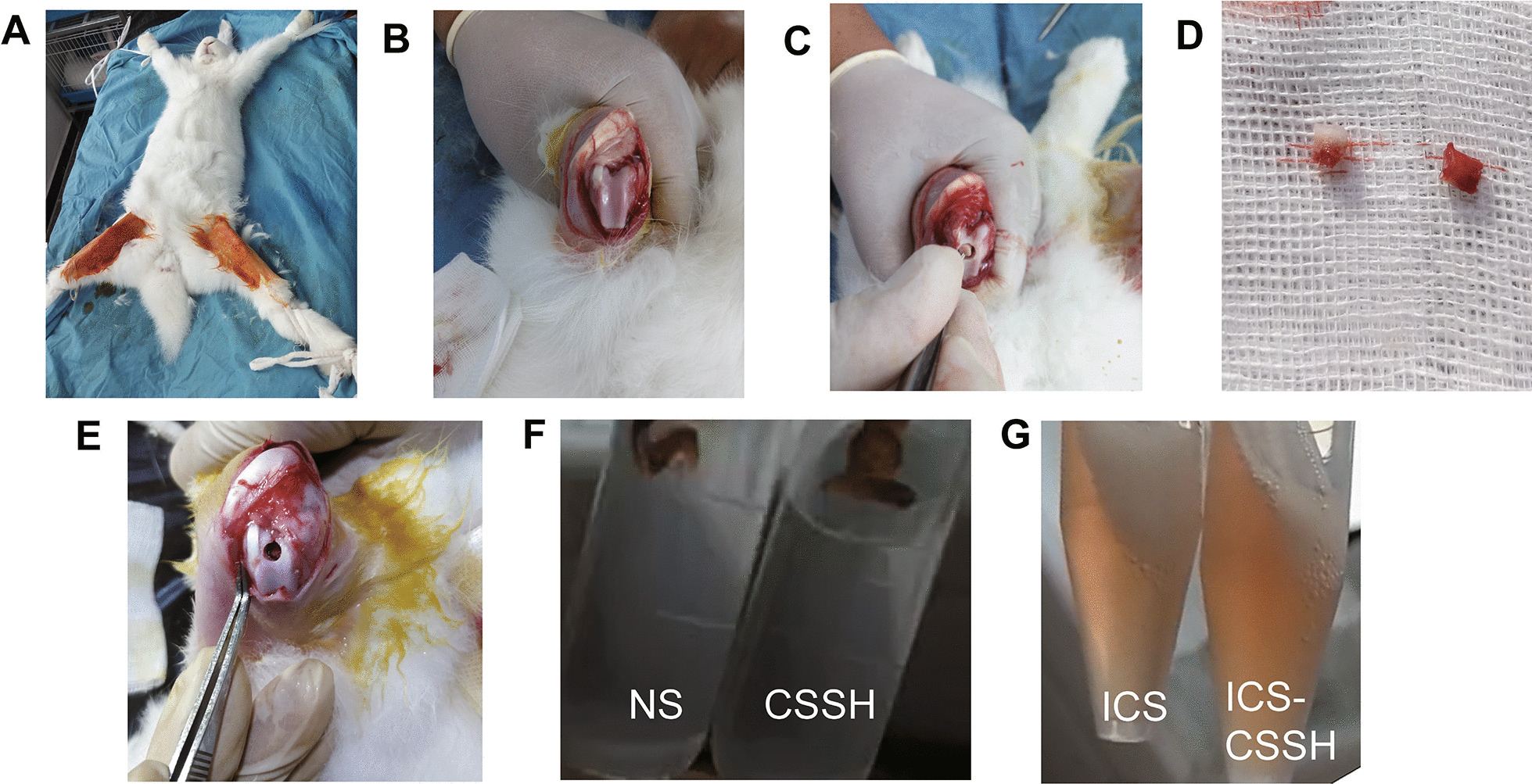
Establishment of osteoarthritis model on rabbits. **A** The rabbit was anesthetized in supine position. **B** The femoral condyle was exposed. **C** The cartilage defect was made on the femoral condyle. **D** The size and depth of the articular cartilage defects was made at a diameter of 4 mm and a depth of 3 mm. **E** The femoral condyle was shown after cartilage defect. **F** The characteristic of normal saline (NS) and chitosan-SH (CSSH) was photoed. **G** The characteristic of icariin-conditioned serum (ICS) and ICS-CSSH was photoed

Rabbits in supine position were anesthetized, and the femoral condyle was exposed for establishment of cartilage defect (Fig. 1A–B). The cartilage defect was made on the femoral condyle (Fig. 1B–C), and the size and depth of the articular cartilage defects was made at a diameter of 4 mm and a depth of 3 mm (Fig. 1D–E). The synthesized CSSH is limpid, and the ICS powder is easily dissolved in normal saline and chitosan solution (Fig. 1F–G).

### ICS-CSSH improved the morphology repair of cartilage defect in vivo

**Fig. 2:**
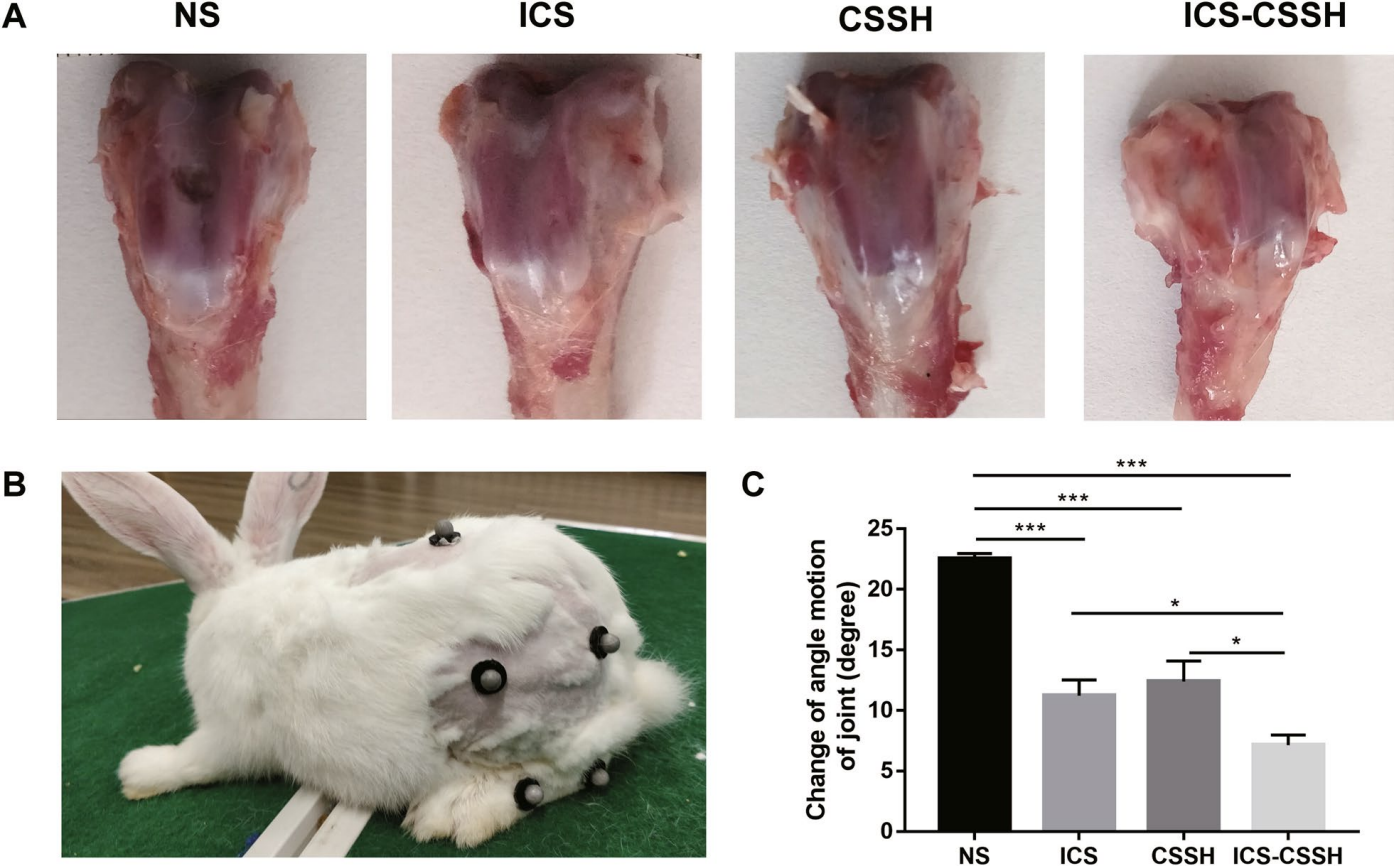
ICS-CSSH improved the repairment of osteochondral defect and positioning performance in rabbit knees. **A** Macroscopic observation of cartilage defect in NS, ICS, CSSH and ICS-CSSH groups. **B** The reflective makers were stuck on the rabbit. The motion data of rabbits were monitored at 200 Hz with a 15-camera motion capture system. **C** Change of angle motion of ankle joint was detected and calculated 12 weeks after surgery (*n* = 6, mean ± SEM). *P < 0.05, ***P < 0.001 versus indicated groups

Twelve weeks after surgery, morphologic changes of osteochondral defect were observed. The rabbits in the NS group showed poor repair of the osteochondral defect, while ICS and CSSH groups showed less cartilage degeneration and defects. The repair in ICS-CSSH group was the most effective, exhibiting less cartilage defects and smooth cartilage surface (Fig. 2A).

### ICS-CSSH promoted positioning performance in rabbit joints

To better evaluate the motor function influenced by osteoarthritis, Vicon motion analysis system was used to detect the positioning performance of rabbits. The reflective makers were stuck on the rabbit. The motion data of rabbits were monitored at 200 Hz with a 15-camera motion capture system before and 12 weeks after surgery (Fig. 2B). Results showed that change of angle motion of ankle joint in NS group was the highest compared with that of treatment groups. As the movement of rabbits in NS group was severely influenced, angle motion of ankle joint was decreased in NS group. As exhibited, change of angle motion of ankle joint in ICS-CSSH group was the lowest exhibiting best recovery of osteochondral defect (Fig. 2C).

### ICS-CSSH improved the repairment of osteochondral defect and subchondral bone in vivo through histologic observation

**Fig. 3:**
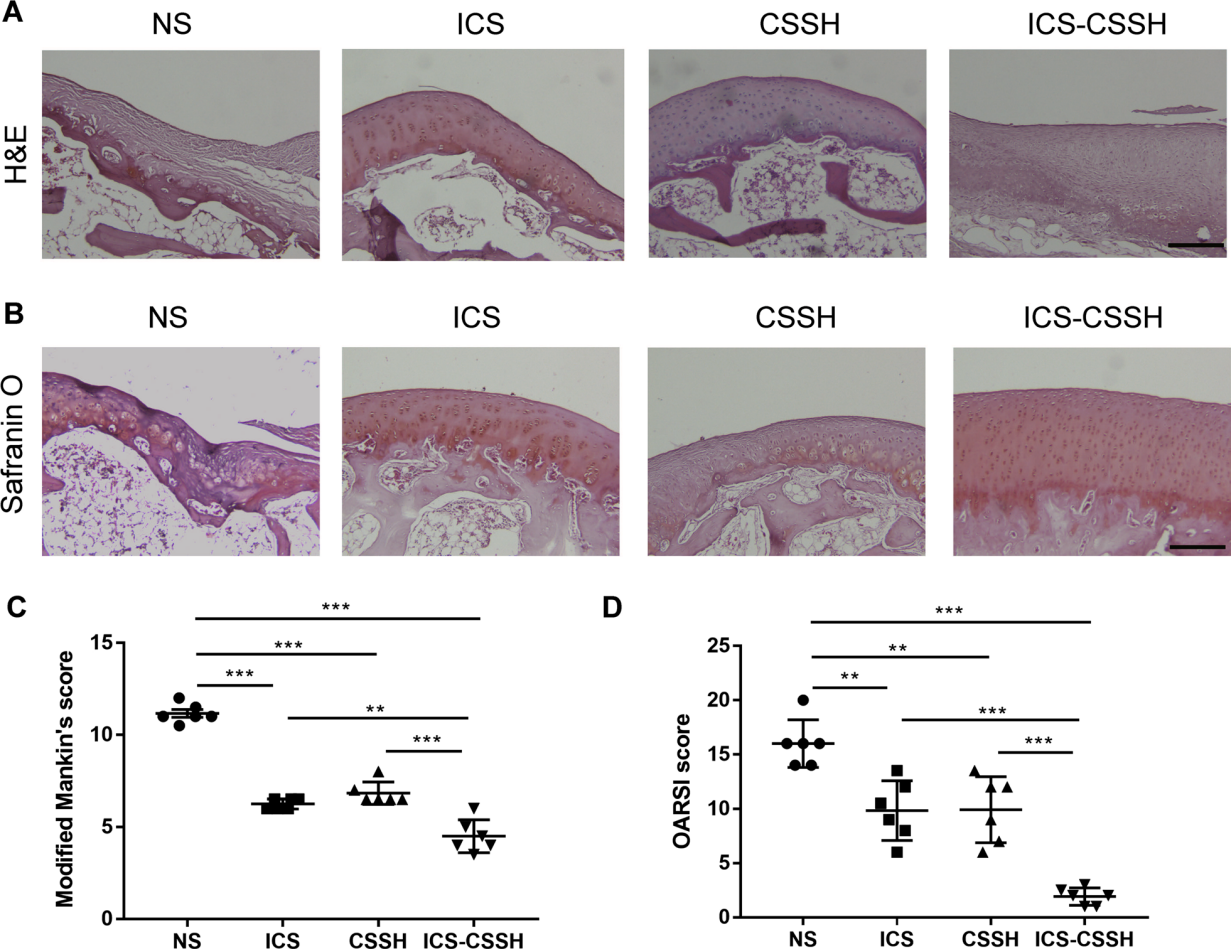
ICS-CSSH improved the repairment of osteochondral defect and subchondral bone in vivo through histologic observation. **A** H&E staining in femoral condyle of rabbit in NS, CSSH, ICS and ICS-CSSH groups. Scale bar, 100 μm. **B** Safranin O staining in femoral condyle of rabbit in NS, CSSH, ICS and ICS-CSSH groups. Scale bar, 100 μm. **C** Modified Mankin’s score for the evaluation of H&E staining in NS, CSSH, ICS and ICS-CSSH groups (*n* = 6, mean ± SEM). **P < 0.01, ***P < 0.001 versus indicated groups. **D** Calculation of Osteoarthritis Research Society International (OARSI) scores in NS, CSSH, ICS and ICS-CSSH groups (*n* = 6, mean ± SEM). **P < 0.01, ***P < 0.001 versus indicated groups

The results of H&E staining and Safranin-O staining showed that ICS-CSSH group had a smoother surface with well-arranged chondrocytes and clear tide lines compared with NS group. In the NS group, the subchondral plate was barely visible and the trabeculae were thinned with cancellous bone loss. The condition of cartilage and subchondral bone in the ICS and CSSH groups was better than that in the NS group but worse than that in the ICS-CSSH group (Fig. 3A–B). Modified Mankin’s score was used for the evaluation of H&E staining in NS, CSSH, ICS and ICS-CSSH groups. It provided a histopathologic correlation with cartilage biochemical changes associated with OA progression. It represented lowest score in ICS-CSSH group in consistent with best repair of osteochondral defect and subchondral bone (Fig. 3C). The defect area was analyzed histologically using the OARSI scores for cartilage regeneration. The OARSI scale evaluation showed a significantly higher score in the treatment groups compared with the NS group (Fig. 3D). Among them, the cartilage repair effect was better in the ICS-CSSH group than in the other treatment groups (Fig. 3D). The higher OARSI score in the ICS-CSSH group showed better recovery of cartilage defects.

### ICS-CSSH promoted the secretion of GAG and collagen II expression in the osteoarthritis knee

**Fig. 4:**
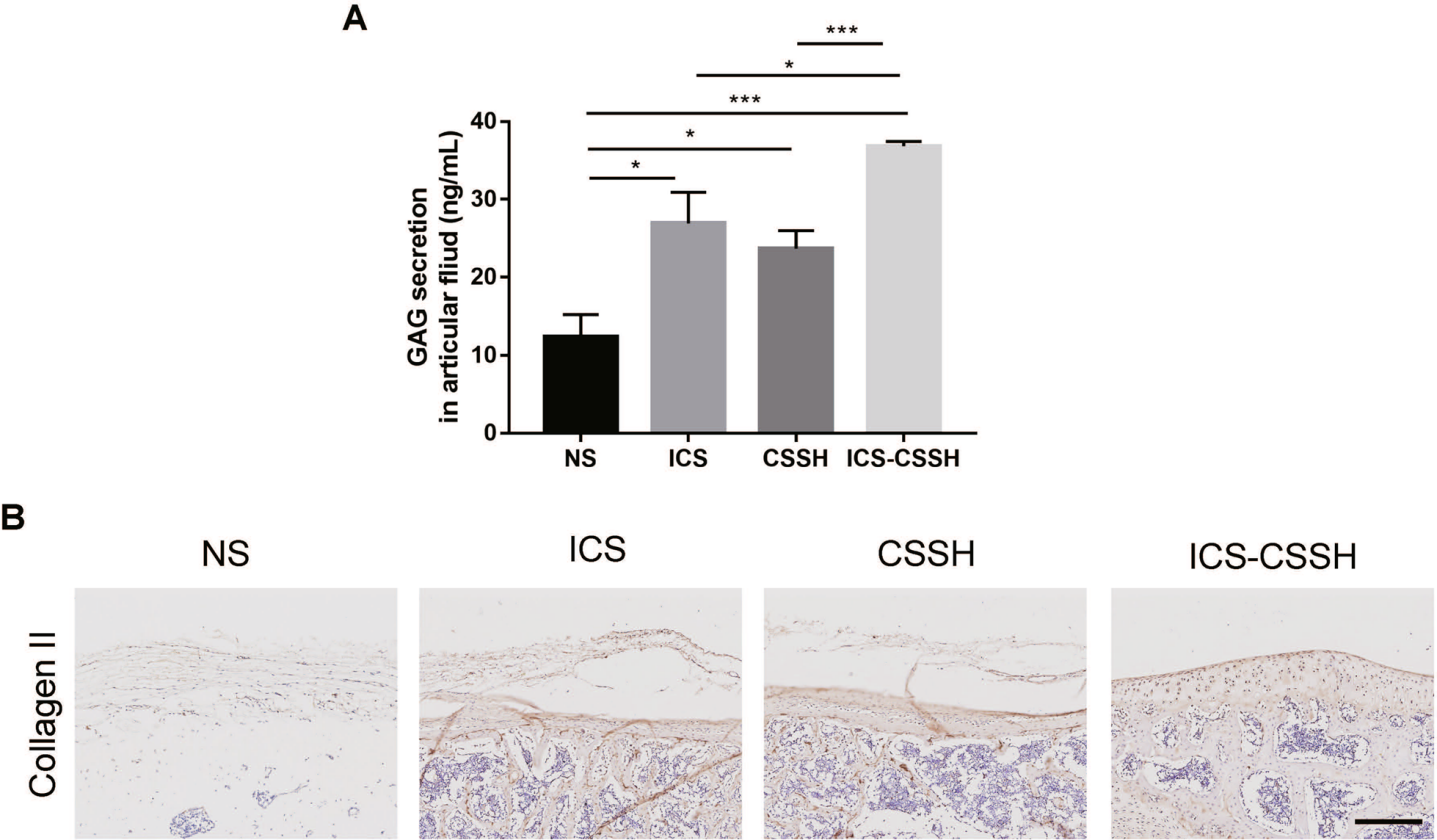
ICS-CSSH promoted the secretion of glycosaminoglycans (GAG) and Collagen II expression in the osteoarthritis knee. **A** ICS-CSSH promoted GAG secretion in the articular fluid (*n* = 6, mean ± SEM). *P < 0.05, ***P < 0.001 versus indicated groups. **B** ICS-CSSH improved Collagen II expression in rabbit knees in vivo through immunohistochemical observation. Scale bar, 100 μm

After surgery for 12 weeks, the joint fluid in the articular cavity was collected and the GAG content was measured. The results showed that the GAG content in the ICS group, CSSH group and ICS-CSSH group was significantly higher than that in the NS group (Fig. 4A). The ICS-CSSH group had higher content than the ICS and CSSH groups. This was some evidence that ICS-CSSH promoted the secretion of GAG in arthritic cartilage and was more effective than ICS or CSSH injections alone.

The function of articular cartilage can be assessed to some extent by immunohistochemistry of type II collagen. After surgery for 12 weeks, fewer and uneven cartilage staining areas in the NS group could be observed, with poorer recovery of cartilage defects. The ICS group was nearly flat and had less positive expression of collagen. In the CSSH group, the cartilage defect repair was uneven, with some cartilage tissue and positive expression of type II collagen. In the ICS + CSSH group, the articular cartilage surface was relatively flat, and the positively expressed type II collagen was uniformly distributed in the repair area (Fig. 4B).

### ICS-CSSH improved mRNA expression of COL2A1, MMP13 and ADAMTS5 in rabbit knees

**Fig. 5:**
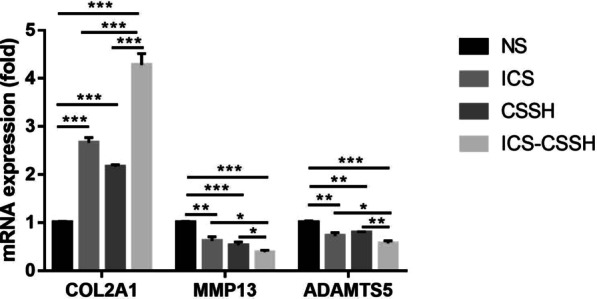
ICS-CSSH improved mRNA expression of COL2A1, MMP13 and ADAMTS5 in rabbit knees (*n* = 3, mean ± SEM). *P < 0.05, **P < 0.01, ***P < 0.001 versus indicated groups

To further evaluate the expression of extracellular matrix related genes in the injured chondrocytes, mRNA expression of COL2A1, MMP13 and ADAMTS5 in rabbit knees was detected. Expression of COL2A1 mRNA was significantly upregulated in ICS-CSSH group, whereas MMP13 and ADAMTS5 downregulated sharply. As extracellular matrix proteins play a key role in the pathogenesis of osteoarthritis, it is indicated that ICS-CSSH promoted the synthesis and secretion of collagen II and inhibited the expression of MMP13, ADAMTS5 (Fig. 5).

## Discussion

KOA is a degenerative condition affecting millions of people worldwide with complex pathogenesis. Besides the breakdown of cartilage, it causes changes in the bone and deterioration of the connective tissues that hold the joint together and attach muscle to bone. Considering this, numerous research has focused on the repair of osteochondral defect and stabilization of subchondral bone. The present research focused on the effects of icariin and the bioactive material chitosan on KOA and pointed out ICS combined with chitosan attenuated cartilage injury and osteoporosis of the subchondral bone in rabbits with KOA.

Due to the avascular property and confinement of resident chondrocytes to extracellular matrix, adult cartilage has a very limited healing capacity after injury. Endeavors have been attempted to rescue the resident chondrocytes and promote the formation of hyaline cartilage in the joint surface. It was also found that subchondral bone is strongly associated with cartilage metabolism [24]. The subchondral bone includes the subchondral bone plate, subchondral trabecular bone and other structures [25]. Unlike cartilage, subchondral bone is rich in nerves, blood vessels, and bone marrow, which play a vital role in nourishing cartilage, maintaining metabolism, and providing shock absorption [3]. Indeed, there is sclerosis of the subchondral bone, thickening of the subchondral plate and an increase in bone mass in the progression of OA [3, 7, 25]. However, in the early stages of KOA, subchondral bone loss was observed, accompanied by cartilage damage. This effect may be caused by abnormal subchondral bone remodeling and cartilage-subchondral bone crosstalk [7, 26]. There is an interaction between cartilage and subchondral bone. It has been shown that degeneration of cartilage leads to loss of subchondral bone in rat models. This was due to the spread of cytokines such as RANKL secreted by chondrocytes into the subchondral bone, leading to proliferation and activation of osteoclasts [27]. Therefore, cartilage and subchondral bone lesions should be considered as a whole in the early stage of KOA. The present study has showed the promotive effects of ICS-CSSH on the formation of hyaline cartilage and repair of subchondral bone. Based on present findings reported here, drug-loaded compatible materials could be considered as a new strategy for the improvement of healing capacity of cartilage and subchondral bone defects.

Icariin has been demonstrated to be capable of enhancing osteogenic differentiation, facilitating matrix calcification and inhibiting osteoclastic bone resorption with or without inductive medium [28–31]. At the same time, studies have indicated that icariin is an effective monomer for the treatment of osteoporosis and has potential clinical value for bone metabolism [32, 33]. This means that icariin is a potential drug that can both repair cartilage and promote subchondral bone remodeling homeostasis. Therefore, icariin could be a potential drug to promote cartilage repair and reduce subchondral bone osteoporosis. In addition, our previous study confirmed that ICA-containing serum is effective in cartilage repair [16].

Chitin is of the most available biopolymers on earth, and chitosan is considered as a highly promising biocompatible biopolymer for numerous applications in the biomedical field (skin, bone, tissue engineering, artificial kidneys, nerves, livers, wound healing). Use of chitosan as a biomaterial or drug delivery agent has recently drawn a considerable attention in the applications for the repair of articular cartilage. Lu et al. have demonstrated that the chitosan solution injected into the knee articular cavity of rats caused a significant increase in the density of chondrocyte in the knee articular cartilage, suggesting that the chitosan could be potentially beneficial to the wound healing of articular cartilage. In addition, a study by Shen et al. found that hydroxyapatite-grafted-chitosan promoted subchondral bone remodeling in arthritic mice and promoted osteogenesis and chondrogenesis [20]. This implies that chitosan can be used as a material for subchondral bone repair and has a synergistic effect on cartilage repair. Given the importance of chitosan in stimulating the chondrogenesis and osteogenesis, chitosan and its derivatives such as CSSH are ideally applied in tissue engineering and osteochondral repair.

In this study, macroscopic observations revealed smoother articular surfaces in the ICS-CSSH group, and after 12 weeks of modeling. The ICS-CSSH group also had better joint mobility and motion ability than the other groups. Histological staining studies revealed irregular cartilage alignment, blurred tide lines, thinning of subchondral bone plates, and cancellous bone loss in early KOA. In contrast, the cartilage layer was more regular after the ICS-CSSH intervention, with clear tide lines and a clear view of the calcified cartilage and subchondral plate. In addition, ICS and CSSH separately also have some repairing effect on cartilage and subchondral bone, but not as well as the ICS-CSSH group.

Hemostasis of extracellular matrix environment plays a key role in the maintenance of cartilage. This consists predominantly of GAGs and collagens supplemented by stabilizing proteins such as link protein, cartilage oligomeric protein, decorin, and fibromodulin. Chitosan easily forms polyelectrolyte complexes with hyaluronan and chondroitin sulfate and develops enhanced performances in regenerating hyaline cartilage, typical results being structural integrity of the hyaline-like neocartilage, and reconstitution of the subchondral bone, with positive cartilage staining for collagen-II and GAG in the treated sites [34]. Importantly, once chondrocytes become hypertrophic, they begin to secrete COL10, ALP, and various MMPs, which work in a concerted effort to initiate bone deposition. Collagen II promoted chondrogenesis while extracellular proteases MMP13, ADAMTS5 facilitated the degradation of extracellular matrix proteins. ADAMTS5 is an aggrecanase that cleaves aggrecan, a major proteoglycan of cartilage, and mediate cartilage destruction in osteoarthritis. As the results shown, the GAG content in the articular fluid of the ICS-CSSH group was also higher than that of the other groups. In the ICS-CSSH group, the expression of collagen II was significantly higher than in the NS group, indicating improved chondrocyte function. Thus, it is promising to use ICS-CSSH in KOA treatment.

Despite extensive research on articular cartilage conducted over the past decades, we still do not have the definitive answer for a successful repair of damaged cartilage and osteoporosis of subchondral bone in KOA. Our research has highlighted that ICS and chitosan-based composites have potential in KOA’s medical applications. Although the positive effects of ICS-CSSH were demonstrated in our study, several limitations existed. It is necessary to expand the limited knowledge of drug-conditioned serum like ICS and further application of chitosan-based composite CSSH. Furthermore, studies should attempt to give the composition of ICS and determine the cytokines and growth factors that might change during the osteochondral defect model. To further promote biomedical tissue engineering in bone research, there is also the need to explore its clinical potential for regeneration of hyaline-like cartilage tissue and stabilization of subchondral bone.

## Conclusion

ICS and CSSH could promote partial integration of cartilage superficial layer and subchondral bone, while ICS-CSSH showed improvement in facilitating formation of osteochondral tissues. ICS combined with CSSH could promote the repair of osteochondral defect, stabilize subchondral bone remodeling and ameliorate subchondral bone osteoporosis in rabbit knees.

## Supplementary Information

**Additional file 1**: Raw data of all figures.

## Data Availability

Raw data were submitted as Additional file 1. Other materials could be provided upon the reasonable request of corresponding authors.

## Abbreviations

KOA: Knee osteoarthritis
ICS: Icariin-conditioned serum
ICS-CSSH: Icariin-conditioned serum with chitosan
OARSI: Osteoarthritis research society international score
IHC: Immunohistochemical
ACS: Autologous conditioned serum
SPF: Specific pathogen free
CSSH: Thiolated chitosan
NS: Normal saline
OD: Optical density
H&E: Hematoxylin and eosin
qPCR: Quantitative real-time PCR
GAG: Glycosaminoglycans
